# Quantitative inference of dynamic regulatory pathways via microarray data

**DOI:** 10.1186/1471-2105-6-44

**Published:** 2005-03-07

**Authors:** Wen-Chieh Chang, Chang-Wei Li, Bor-Sen Chen

**Affiliations:** 1 Lab. of System Biology, National Tsing Hua University, 101, Sec 2, Kuang Fu Road, Hsinchu, 300, Taiwan; 2 Department of Electrical Engineering, National Tsing Hua University, 101, Sec 2, Kuang Fu Road, Hsinchu, 300, Taiwan

## Abstract

**Background:**

The cellular signaling pathway (network) is one of the main topics of organismic investigations. The intracellular interactions between genes in a signaling pathway are considered as the foundation of functional genomics. Thus, what genes and how much they influence each other through transcriptional binding or physical interactions are essential problems. Under the synchronous measures of gene expression via a microarray chip, an amount of dynamic information is embedded and remains to be discovered. Using a systematically dynamic modeling approach, we explore the causal relationship among genes in cellular signaling pathways from the system biology approach.

**Results:**

In this study, a second-order dynamic model is developed to describe the regulatory mechanism of a target gene from the upstream causality point of view. From the expression profile and dynamic model of a target gene, we can estimate its upstream regulatory function. According to this upstream regulatory function, we would deduce the upstream regulatory genes with their regulatory abilities and activation delays, and then link up a regulatory pathway. Iteratively, these regulatory genes are considered as target genes to trace back their upstream regulatory genes. Then we could construct the regulatory pathway (or network) to the genome wide. In short, we can infer the genetic regulatory pathways from gene-expression profiles quantitatively, which can confirm some doubted paths or seek some unknown paths in a regulatory pathway (network). Finally, the proposed approach is validated by randomly reshuffling the time order of microarray data.

**Conclusion:**

We focus our algorithm on the inference of regulatory abilities of the identified causal genes, and how much delay before they regulate the downstream genes. With this information, a regulatory pathway would be built up using microarray data. In the present study, two signaling pathways, i.e. circadian regulatory pathway in *Arabidopsis thaliana *and metabolic shift pathway from fermentation to respiration in yeast *Saccharomyces cerevisiae*, are reconstructed using microarray data to evaluate the performance of our proposed method. In the circadian regulatory pathway, we identified mainly the interactions between the biological clock and the photoperiodic genes consistent with the known regulatory mechanisms. We also discovered the now less-known regulations between crytochrome and phytochrome. In the metabolic shift pathway, the casual relationship of enzymatic genes could be detected properly.

## Background

Biological phenomena at different organismic levels have revealed some sophisticated systematic architectures of cellular and physiological activities implicitly. These architectures were built upon the biochemical processes before the emergence of proteome and transcriptome [1-3]. Under the molecular machinery, the biochemical processes are mostly interpreted as frameworks of connectivity between biochemical compounds and proteins, which are synthesized from genes to function as transcription factors binding to regulatory sites of other genes, as enzymes catalyzing metabolic reactions, or as components of signal transduction pathways [4-6]. This implies that, in order to understand the molecular mechanism of genes in the control of intracellular or intercellular processes, the scope should be broadened from DNA sequences coding for proteins to the systems of genetic regulatory pathways determining which genes are expressed, when and where in the organism and to which extent [7]. In the experience of engineering field, the systematic architecture and dynamic model could investigate the characteristics of signaling regulatory pathways [8]. Therefore, how to construct the dynamic model of a signaling pathway from the system structure point of view might be the first key to the door of system biology. Most biological phenomena directly or indirectly influenced by genes such as metabolism, stress response, and cell cycle are well studied on the molecular basis. Thus, identification of a signal transduction pathway could be traced back to the genetic regulatory level. The rapid advances of genome sequencing and DNA microarray technology make possible the quantitative analysis of signaling pathway besides the qualitative analysis. More particularly, the embedded time-course feature of microarray data would promote the system analysis of signal regulatory pathways as well, which is very mature in the field of engineering.

In addition to northern blots and reverse transcription-polymerase chain reaction (RT-PCR), which study a small number of genes in a single assay, the transcriptome analysis has, via DNA microarray technology [9], managed to achieve high-throughput monitoring of the almost genome-wide mRNA expression levels in living cells or tissues. Two types of available microarrays, the spotted cDNA and *in situ *synthesized oligonucletide [10] chips, which permit the spatiotemporal expression levels of genes to be rapidly measured in a massively parallel way, are used in different experimental requirements and stocked in the databases on net, such as Stanford Microarray Database (SMD) [11], Gene Expression Omnibus(GEO) [12] in NCBI, and ArrayExpress [13] in EBI. Microarray experiments are now routinely used to collect large-scale time series data that facilitate quantitative genetic regulatory analysis while qualitative discussion is the traditional thinking [14-17].

Several analytic methods have been proposed to infer genetic interrelations from gene expression data. In the coarse-scale approach of clustering, the underlying conjecture is that co-expression is indicative of the co-regulation, thus clustering may identify genes that have similar functions or are involved in the related biological processes. The most widely used method is the unsupervised hierarchical clustering [18]. This approach has an increasing number of nested classes by similarity measurement and resembles a phylogenetic classification. If we know the number of clusters in advance, the k-means clustering [19] could assign gene elements into a fixed number k of clusters in a way to minimize the overall Pearson or Euclidean distances of each member internally in the same cluster. Other algorithms such as the neural-network-based self-organizing maps (SOM) [20], singular value decomposition (SVD) or principal component analysis (PCA) [21], and fuzzy clustering methods [19] also have their own advantages and limitations. Alternative supervised clustering algorithm of support vector machine [22], which uses prior biological information of cluster for training, would enhance the accuracy of clustering. However, the nature of clustering algorithms apparently cannot uncover the causal interactions between genes just by grouping. Regarding the causality of pathways, the clustering analysis needs to cooperate with sequence motif detection [23]. It is also important to note that models using clustering analysis are static and thus can not describe the dynamic evolution of gene expression, even in the type of time-course microarray data.

A statistical model of Bayesian network [24] was proposed to model genetic regulatory networks. Basically, the technique uses a probabilistic score to evaluate the networks with respect to the expression data and searches for the network with the optimal score. The dynamic Bayesian network [25] was proposed to learn the network structure and parameters by maximizing the posterior probability via Bayes rule of prior probability and marginal likelihood. Another algorithm of Boolean networks [26] can also be employed to model the dynamic evolution of gene expression, where the state of a gene can be simplified to being either active (on, 1) or inactive (off, 0). The probabilistic nature of Bayesian networks is capable of handling noise inherent in both the biological processes and the microarray experiments. This makes Bayesian networks superior to Boolean networks, which are deterministic in nature. The validity of dynamic Bayesian networks is evaluated by the sensitivity-specificity score ratios [25], which depend on the training size, the degree of accuracy of prior assumption. A genetic regulatory network based on the first order differential equation with given decay rates was discussed in [27].

In this study, the dynamic system approach could be employed to model how a target gene's expression profile is regulated by its upstream regulatory genes from the system causality point of view. Then, with the causal dynamic model, the upstream regulatory function can be extracted from the expression profile of the target gene by the optimal estimation method, i.e. maximum likelihood estimation. Since merely the second-order differential equation is employed to model the dynamic evolution of the target gene, only a few parameters need to be estimated. Furthermore, the derived regulatory function is closely related to the causal upstream information of the pathway and will create a basis for inferring the regulatory pathway from the system biology point of view.

In either eukaryote or prokaryote, signaling regulatory pathways are considered as responses to the physiological activities or the deviation from homeostasis, which would affect the normal states of an organism. Among these signaling regulatory pathways, cell cycle [17] is one of the most conspicuous features of life which plays an important role in growth and cellular differentiation in all organisms. In plants, the stress-induced pathways [28] are very important to survivability under the abiotic environmental treatment such as drought, salinity and cold[29]. If these critical pathways can be identified from quantitative analysis *in silico*, the defect of biological processes would be predicted and corrected before hand. Our aim is to construct signaling regulatory pathways quantitatively by the system inference approach with a dynamic model and microarray data.

In this study, a second-order differential equation, which has been widely used to model many physical dynamic systems with good characteristics, is proposed to model the time-profile evolutional behavior of a target gene. The regulatory function is taken as the driving input of the dynamic equation of the target gene. Using the dynamic equation and microarray data, we first extract the regulatory function for each target gene. According to the extracted regulatory function, we deduce their upstream regulators to trace back upstream signaling pathways. Then, upstream regulatory genes are taken as target genes to trace back their upstream regulatory genes. Iteratively, we can construct the whole regulatory pathway to the genome wide using the dynamic regulatory model and microarray data from the system biology point of view. Finally, we give some independent validation of our approach by repeating the analysis with randomly reshuffling the time order of microarray data and see if the proposed pathways are destroyed.

We have applied our dynamic system approach to two genetic regulatory pathways with microarray data sets publicly available on net [15,30]. One is the circadian regulatory pathway in *Arabidopsis thaliana *[31,32], and the other is the metabolic shift pathway from fermentation to respiration in yeast *Saccharomyces cerevisiae *[33]. The circadian system is an essential signaling pathway that allows organisms to adjust cellular and physiological processes in anticipation of periodic changes of light in the environment [34-38]. According to the synchronously dynamic evolution of microarray data, we have successively identified the core signaling transduction from light receptors to the endogenous biological clock [39,40], which is coupled to control the correlatively physiological activity with paces on a daily basis. On the other hand, the diauxic shift [41] from the exhausted fermentable sugar of anaerobic metabolism to aerobic growth is correlated with widespread changes in the expression of genes involved in fundamental cellular processes such as carbon metabolism, protein synthesis, and carbohydrate storage. [28,31,32,42-47] The architecture of the signaling pathway correlative to glycolysis or gluconeogenesis during the diauxic shift is properly built up. With the dynamic system approach, not only the regulatory abilities between causal genes could be derived, but also the delays of regulatory activity are specified. These quantitative characteristics will help determine the intrinsic frameworks of connectivity in the above interesting pathways from the system biology point of view.

## Results

The proposed methods in this study would be divided into four steps. In the first step, a dynamic model using the second-order differential equation is developed to describe the expression profile data as output and the regulatory function as input to denote the implicit characteristics of each gene with some parameters. With the help of the second-order dynamic model, we would then extract the upstream regulatory function from the expression profile of the target gene using the optimal estimation method. In the third step, the regulatory function estimated will help seek the correlative regulatory signals from the upstream paths. Iteratively, we can reconstruct the whole signaling regulatory pathway by linking up the upstream regulatory paths. Finally, some biological filters using available biological knowledge are employed to prune the constructed signaling regulatory pathway to improve the accuracy of the proposed method.

### I. Dynamic system description of signaling regulatory model

The second-order differential equation is well used in the description of dynamic system evolved from the causality of gene regulatory function. Let *X*_*i *_(*t*) denote the expression profile of the *i*-th gene at time point *t*. The following second-order differential equation is proposed to model the expression level of the *i*-th gene,



where *G*_*i*_(*t*) is the upstream regulatory function to influence the expression profile *X*_*i*_(*t*) of the *i*-th gene while *a*_*i*_, and *b*_*i*_are the parameters that characterize the dynamic inherent property of the gene like degradation and oscillation, and *ε*_*i*_(*t*) is the noise of current microarray data or the residue of the model. In general, the second-order differential equation has been widely used to model dynamic systems to characterize efficiently the dynamic properties of damping and resonance of systems in physics and engineering.

Obviously, the clue of upstream regulatory pathways is in *G*_*i*_(*t*). Thus, the first step is to detect the upstream regulatory function *G*_*i*_(*t*) from both dynamic equation in (1.1) and microarray data. However, to detect the input regulatory function *G*_*i*_(*t*) from both equation (1.1) and microarray data directly is not easy. In this situation, a Fourier decomposition technique is employed to decompose *G*_*i*_(*t*) as a synthesis of some harmonic sinusoid functions so that the signal detection problem of *G*_*i*_(*t*) is reduced to a simple parameter estimation problem.

Accordingly, we can decompose *G*_*i*_(*t*) by the following Fourier series,



Then the detection of *G*_*i*_(*t*) becomes how to estimate the Fourier coefficients of *α*_*n *_and *β*_*n*_, which are the magnitudes of different harmonics of cos(*nωt*) and sin(*nωt*), for *n *= 0,..., *N *in equation (1.2), respectively. In science and engineering, the Fourier series has been widely employed to synthesize any continuous functions with finite energy. The estimation of *α*_*n*_, *β*_*n *_and the detection of *G*_*i*_(*t*) in equation (1.2) are given in Methods in the sequel.

As a result of parameter estimation in Methods, the detection  of regulatory function *G*_*i*_(*t*) could be derived as follows,



Since the input regulatory function *G*_*i*_(*t*) of a target gene is usually due to the transcriptional binding or some physical interactions from the upstream regulatory genes, in the following, we would trace back to the corresponding regulatory genes from input regulatory function  of the target gene.

### II. Inference of the regulatory pathway via 

Apparently the input regulatory function *G*_*i*_(*t*) in equation (1.1) contains the driving information for the target gene's expression from the upstream regulatory genes. The identified regulatory function  from equation (1.3) could be interpreted as the regulatory connectivity through transcriptional binding or protein-protein interaction imposed on the *i*-th target gene. Nevertheless, the expression data of protein type which should be considered directly in practice are by now unavailable and unreliable to trace back upstream regulatory genes. Instead, the expression data on mRNA level which is now widely available from microarray assays would make tracing back the upstream regulatory pathway possible under proper assumptions. All along the paper we assume that the expression levels of mRNA transcripts are proportional to the actual number of corresponding proteins in the cell. This assumption is indeed a strong approximation since post-transcription is known to play a very important role in down regulating the number of the transcription factor in the cell.

Before the inference of upstream regulatory genes, it is reasonable to confine the effect of the regulatory genes on the regulated target gene. The saturated activity of expression level reveals that the regulatory ability cannot extend unlimitedly. The sigmoid function is often chosen to express the nonlinear saturation with proper parameters. Here, we apply the sigmoid transformation to represent the 'on' and 'off activities of the regulatory genes on binding or not to motifs of the target gene. So the regulatory signal  shown below with the parameter set of *θ*_*j *_= {*γ*, *M*_*j*_, *τ*_*j*_} is the sigmoid transformation of *X*_*j*_(*t*), the expression profile of the *j*-th regulatory gene.



where *γ *is the transition rate, *M*_*j *_is the mean expression of the *j*-th regulatory gene's profile, and *τ*_*j *_is the corresponding signal transduction delay.

The delay activity should be considered in order to describe the signal transduction delay *τ*_*j *_from the *j*-th regulatory gene to the target gene. The delay *τ*_*j *_would be computed by statistical correlation between the regulatory signal  transformed from the *j*-th regulatory gene and the identified regulatory function  of the target gene. The delay *τ*_*j *_is determined by the following maximum correlation criterion,



where *r*_*τ *_is the correlation between  and  under variable delay *τ*. If there are many *τ *to achieve the maximum correlation in (2.2), then only the smallest one is chosen.

Using the correlation method, we trace back *R*_*i *_regulatory genes whose regulatory signals  are most correlated with the regulatory function  of the *i*th target gene, i.e. choose *R*_*i *_genes with maximum correlation but with smaller *τ*_*j *_in (2.2). The determination of number *R*_*i *_will be discussed later. Then, we construct the regulatory pathway by tracing back *R*_*i *_regulatory genes from the identified regulatory function  of the target gene as the following kinetic relationship,



where *c*_*ij *_is the pathway kinetic parameters from the regulatory gene *j *to the target gene *i*, *R*_*i *_are the searched upstream regulatory genes, the constant *c*_*i*0 _represents the basal level to denote the regulatory function other than upstream regulatory genes, and *e*_*i*_(*t*) is the residue of the model.

Furthermore, to estimate the pathway kinetic parameters *c*_*ij*_, equation (2.3) for *m *time points should be written in the following regression form,



where , .

We assume that each element in the error vector, *e*_*i*_(*t*_*k*_), *k *= {1,..., *m*}, is an independent random variable with a normal distribution with zero mean and variance *σ*^2^. By maximum likelihood parameters estimation method (see Methods), the estimates of *σ*^2 ^and Ω_*i *_are given as follows, which is solved as



and



It should be noted that with the combination of biological knowledge about the transcriptional factors, protein phosphorylation, post-transcriptional and specific enzyme regulation of target genes, lots of putative and verified genes correlated with the target genes are pruned by this biological filter for the more efficient and accurate searching of *R*_*i *_upstream regulatory genes in equation (2.3). For example, suppose the expression profile of gene j has a high correlation with regulation function *G*_*i*_(*t*) of target gene i. However, if gene j is not a transcription factor, protein phosphorylation, post-transcription or specific enzyme of target gene i, it will be deleted from the candidates of *R*_*i *_upstream regulatory genes because it may be only a co-expressed gene with the target gene corregulated by the other gene. On the contrary, a verified regulatory gene should be recruited into the candidates even with small correlation with *G*_*i*_(*t*).

Finally, we take the well-known Akaike Information Criterion (AIC) into account for determining the number *R*_*i *_of regulatory signal [42],



The first term in AIC is the residual variance and the second term *R*_*i *_is the number of regulatory genes. AIC includes both the estimated residual variance and model complexity in one statistic, which decreases as *σ*^2 ^decreases and increases as *R*_*i *_increases. AIC has been widely employed to determine the complexity of system modeling science and engineering [42]. The optimal number *R*_*i *_of the upstream regulatory genes will be determined by the minimization of the *AIC *value in equation(2.7).

**Figure 1:**
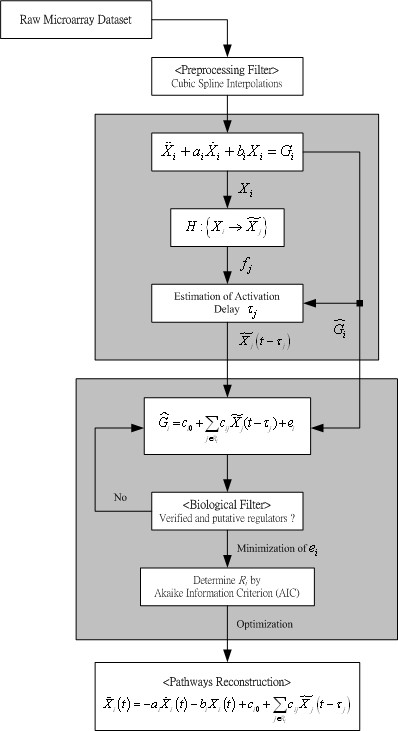
Illustration of the overall flowchart of the pathway inference algorithm.

Now, for the selected target genes in the interesting pathway, we could search for the optimal *R*_*i *_upstream regulatory genes by AIC in equation (2.7) after the biological filtering and determine their pathway kinetic parameters *c*_*ij *_of regulatory signal by equation (2.6). After biological filter pruning, if the number of candidates of regulatory genes is still less than *R*_*i *_determined by AIC, then some genes, which are highly correlative to *G*_*i*_(*t*) but not of transcription factors or signaling proteins of target gene i, should be recruit into candidates to uncover regulatory relationships that were not suspected to be connected. After the combination of equations (2.3) and (1.1), the whole regulatory pathway is obtained as



for *i *= {l, 2,..., *L*}, and *L *is the number of target genes in the pathway. The sub-paths related to the *i*-th target gene in the interesting pathway could be detected by the inference algorithm. Then, it is natural that the whole regulatory pathway would be constructed by the links of all the sub-paths. We also outline the whole flowchart of our dynamic inferring algorithm as shown in Figure 1 for an overview.

## Discussion

### Data set of analysis

The two famous modeling organisms, *Arabidopsis thaliana *and yeast *Saccharomyces cerevisiae*, have been well studied biologically and their microarray assays are abundant. Thus, we chose different types of pathways, one is the plant behavior under environmental variation and the other is the cellular metabolism in response to exhaustion of external source, as examples in this study. In other words, two signaling pathways, i.e. circadian regulatory pathway in *Arabidopsis thaliana *and metabolic shift pathway from fermentation to respiration in yeast *Saccharomyces cerevisiae*, are constructed from microarray data to confirm the accuracy of our proposed method.

For cells grown in the light/dark cycle according to circadian rhythm, Harmer and colleagues [15] used highly reproducible oligonucleotide-based arrays representing about 8200 different genes to determine steady-state mRNA levels in *Arabidopsis thaliana *that are measured in replicate hybridization of 12 samples harvested every 4 hours over 2 days. With their investigation on the circadian regulatory system, Harmer et al. have provided an abundance of correlated genes for the regulatory pathway inference.

As for the metabolic pathway, an cDNA microarray assay from DeRisi et al. [30], containing approximately 6400 distinct expression sequence tags (ESTs) in yeast *Saccharomyces cerevisiae*, is harvested at seven successive 2-hour intervals after an initial nine hours of growth under the diauxic shift. Adoption of the diauxic shift data set would make possible the inference of metabolic shift pathways.

### Process of raw microarray data

**Figure 2:**
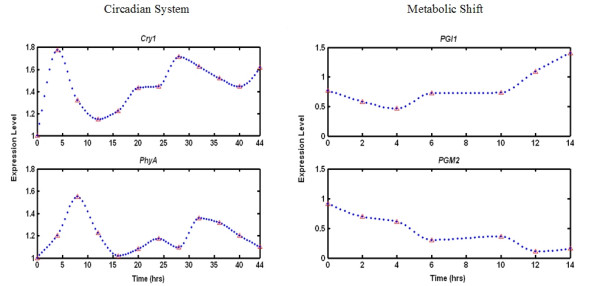
**The expression profiles derived from the cubic spline interpolations**. The expression profiles after performing the cubic spline interpolations for *Cry1*, *PhyA *(pathway of circadian regulatory system of *Arabidopsis thaliana*) on the left-hand side, and *PGI1*, *PGM2 *(metabolic shift pathway of yeast *Saccharomyces cerevisiae*) on the right-hand side. The red open triangles are the raw microarray data, and the blue dotted points are the interpolation data.

With the second-order equation and the optimal estimation method, the dynamic model should be developed first for the regulatory scheme of target genes in the signaling regulatory pathway. Because the raw microarray data sample of the biological assays that will be analyzed is small with less than 15 data points for an individual gene, the cubic spline method is used to interpolate the observed data to increase the data points of each gene's time-course microarray data. As shown in Figure 2, the expression profiles of *Cry1 *(CRYTOCHROME 1) and *PhyA *(PHYTOCHROME A) genes in the circadian regulatory pathway of *Arabidopsis thaliana *are interpolated by the cubic spline method among raw data points on the left-hand side. Similarly, *Pgi1 *(PHOSPHOGLUCOSE ISOMERASE 1) and *Pgm2 *(PHOSPHOGLUCOMUTASE 2) genes in the metabolic shift pathway of yeast *Saccharomyces cerevisiae *are on the right-hand side. After the expression profiles are smoothed by the cubic spline technique, we can obtain the data of the first derivative  and the second derivative  more accurately and abundantly.

### Extraction of regulatory information

**Figure 3:**
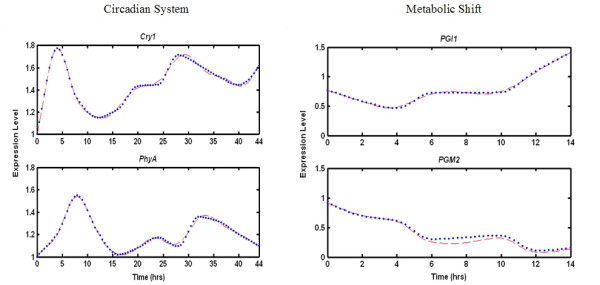
**The second-order dynamic model fitting of pathway genes**. The expression profile with corresponding second-order dynamic model fitting for *Cry1*, *PhyA *(pathway of circadian regulatory system of *Arabidopsis thaliana*) on the left-hand side, and *PGI1*, *PGM2 *(metabolic shift pathway of yeast *Saccharomyces cerevisiae*) on the right-hand side. The blue dotted points are the cubic spline interpolations of microarray data, and the red dashed lines are the estimated dynamic evolution of expression data.

**Figure 4:**
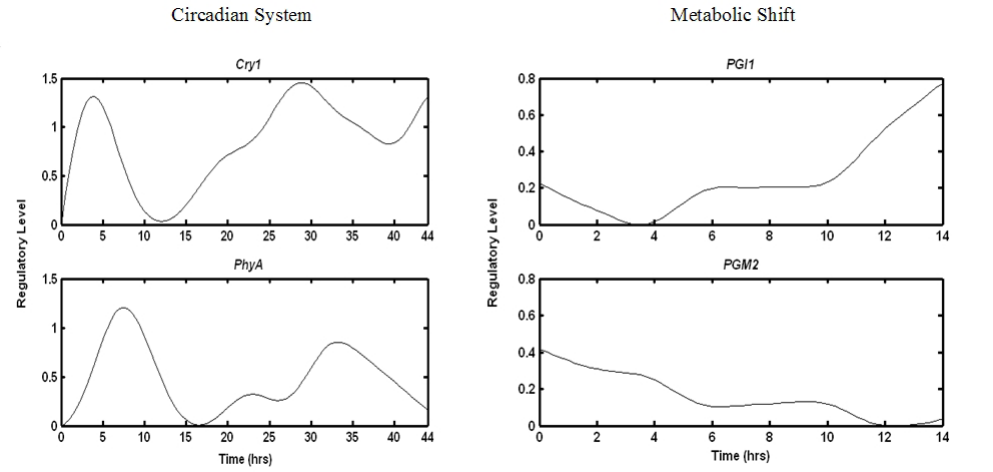
**The extracted upstream regulatory functions of pathway genes**. The upstream regulatory function  extracted from expression profiles of corresponding target genes *Cry1*, *PhyA *(pathway of circadian regulatory system of *Arabidopsis thaliana*) on the left-hand side, and *PGI1*, *PGM2 *(metabolic shift pathway of yeast *Saccharomyces cerevisiae) *on the right-hand side.

After data expansion by the cubic spline method, we would have enough data to estimate the parameters of the regulatory dynamic model of the target gene from equation (2.4). Following the dynamic model in equation (3.1), the parameters which characterize the dynamic regulatory mechanism are estimated successfully for each target gene in the pathway. By dynamic model fitting, the expression profiles of the mentioned genes in Figure 2 can be reconstructed in Figure 3 with time progression again. Hence, we not only could predict the dynamic evolution of the target gene's expression profile accurately, but also deduce the regulatory function  simultaneously as the scheme of Figure 4. The regulatory information between target genes and their upstream genes can be extracted properly with this method.

### Inference of the regulatory pathway

**Figure 5:**
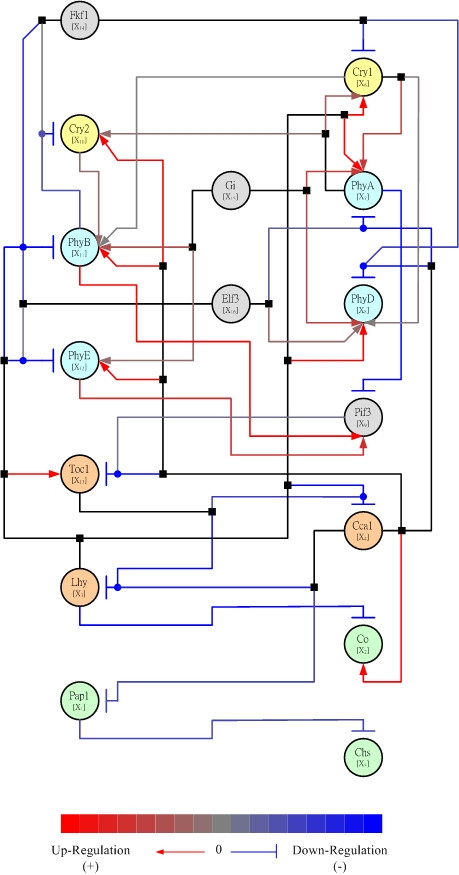
**The pathway of circadian regulatory system of Arabidopsis thaliana according to the dynamic regulatory modeling in Table 1**. The related genes are represented as ovals with different colors of light yellow (crytochrome), light blue (phytochrome), orange (biological clock genes), light green (some physiologically light-dependent downstream genes), and gray (other relevant genes not as target genes). There are three types of lines with colors of red (activation), blue (repression), and black. In addition to the black line representing the signal pipes from genes, the red lines are shown depending on the degree of activation whilst the blue lines are shown depending on the degree of repression. The black square symbol attached on the lines is the bifurcate node from the same pipe of signal. The circle symbol attached on the lines is the collecting nodes from different signal sources. The colored degree bar between activation and repression is also shown in the bottom of the figure.

**Figure 6:**
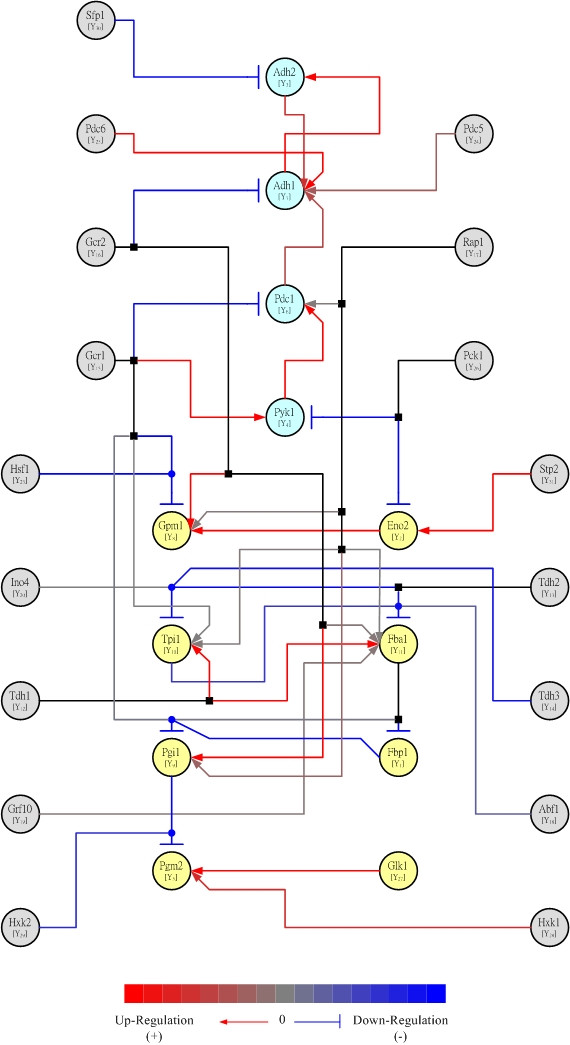
**The metabolic shift pathway of yeast Saccharomyces cerevisiae according to the dynamic regulatory modeling in Table 2**. The related genes are represented as ovals with different colors of light blue (fermentation), light yellow (gluconeogenesis), and gray (other relevant genes not as target genes). There are three types of lines with colors of red (activation), blue (repression), and black. In addition to the black line representing the signal pipes from genes, the red lines are shown depending on the degree of activation whilst the blue lines are shown depending on the degree of repression. The black square symbol attached on the lines is the bifurcate node from the same signal source. And the circle symbol attached on the lines is the collecting nodes from different signal sources. The colored degree bar between activation and repression is also shown in the bottom of the figure.

For illustrations, the inferring strategy is applied to the selected core genes (X_1_~X_13 _and Y_1_~Y_11_) in two pathways of the circadian regulatory system in *Arabidopsis thaliana *and the metabolic shift pathway in yeast *Saccharomyces cerevisiae *to recognize their upstream regulatory genes, respectively. Their regulatory abilities with signal transduction delays are shown in the form of dynamic equation in Table 1 and Table 2, respectively. These regulatory abilities implying different degrees of influence are converted into a red-colored line as positive regulation (activation) and a blue-colored line as negative regulation (inhibition) for each target gene. Then, according to the dynamic regulatory equations in Table 1 and Table 2, the pathways of the circadian regulatory system and the metabolic shift pathway are described in Figure 5 and Figure 6, respectively.

#### a. Pathway of circadian regulatory system

The circadian rhythm controls processes ranging from cyanobacteria cell division to human wake-sleep cycles. In plant, especially for *Arabidopsis thaliana*, the growth and development have adapted to the diurnal cycling of light and dark [28,31,32,42,44,46-49]. The ability of plants to respond to light is achieved through some photoreceptors. Two classes of photoreceptors are well known to form the photo-transduction pathway under the circadian regulatory system in *Arabidopsis thaliana *[50]. One is the crytochrome of blue-light photoreceptors, containing *Cry1 *and *Cry2*. The other is the phytochrome of mainly red-light photoreceptors, including *PhyA*, *PhyB*, *PhyD *and *PhyE*.

In the photo-transduction related genes (Table 1 and Figure 5), containing both crytochrome (*Cry1 *and *Cry2*) and phytochrome *(PhyA, PhyB, PhyD *and *PhyE*)*, Cry1 *[X_6_] and *Cry2 *[X_10_] are commonly regulated by *Lhy *[X_3_] (LATE ELONGATED HYPOCOTYL) in reciprocal ways with significant values (0.7569 in Eq.(6) and -1.8773, Eq.(10) of Table 1, respectively), implying the essentially regulatory role of *Lhy *on crytochrome genes. In addition, from Eq.(10) in Table 1, we further observe that *Ccal *[X_4_] (CIRCADIAN CLOCK ASSOCIATED 1) has the greatest positive regulation (2.3465) on *Cry2*, meaning that *Cry2 *is jointly regulated by *Lhy *and *Ccal*. Because the binding sites of *Lhy *and *Ccal *found in the promoter regions of *Cry2 *[51] are consistent with our inference, the transcriptional binding might be the mechanism of *Cry2 *affected by both *Lhy *and *Ccal*. In addition, the mutual activations of phosphorylation between *Cry1 *[X_6_] and *PhyA *[X_7_] in Eq.(6) and Eq.(7) of Table 1 are specifically identified consistent with the previous work [52]. At present, little is known about the nature of interactions between these two classes of photoreceptors. From Eq.(10) in Table 1, *Cry2 *[X_10_] is also positively regulated by *PhyA *[X_7_] with 0.5-hr activation delay similar to that in *Cry1 *(Eq.(6) in Table 1). Therefore, *PhyA *is considered as a post-transcriptional regulator of phosphorylation to crytochrome within 1.0-hr after transcription. On the other hand, *PhyB *[X_11_] down-regulates *Cry2 *with a significant effect (-0.7141) while *Cry2 *[X_10_] up-regulates *PhyB *(0.0511) weakly by feedback (see Eqs.(10), (11) in Table 1.). The mutual interactions between *Cry2 *and *PhyB *in nuclear speckles that are formed in a light-dependent fashion are also confirmed by Mas *et al*. [48]. Because *Cry1 *and *Cry2 *are both negatively co-regulated by *PhyD *[X_8_] and *PhyE *[X_12_] significantly (see Eqs.(6), (10) in Table 1), *PhyA *has apparently different behavior from *PhyB*, *PhyD*, and *PhyE *in activating crytochrome. This might suggest the mechanism that *PhyA *mediates the blue light by up-regulating *Cry1 *and *Cry2*, whilst *PhyB*, *PhyD*, and *PhyE *would mediate the red light by inhibiting blue photoreceptors [53,54].

In the mainly red-light photoreceptors of phytochrome *(PhyA, PhyB, PhyD *and *PhyE) *in Figure 5, undoubtedly *Lhy *[X_3_] and *Ccal *[X_4_], well-known biological clock genes in the circadian system [40,46], are core regulators involved in the transcriptions of both phytochrome (see Eqs.(7), (8), (11), and (12) in Table 1) and crytochrome (see Eqs.(6), (10) in Table 1) via feedback transcriptional binding. Similarly, *Gi *[X_15_] (GIGANTEA) in Figure 5 has been identified as a manifested regulator to all the phytochromes (also see Eqs.(7), (8), (11), and (12) in Table 1), although *Gi *sequence lacks any motifs suggesting that it is a transcription factor of phytochromes [55]. Hence, *Gi *might be a post-transcriptional regulatory factor. However, there is another gene *Elf3 *[X_16_] (EARLY FLOWERING 3) opposite to *Gi *on phytochrome, especially for *PhyA, PhyB *and *PhyE *(Eqs.(7), (11) and (12) in Table 1). Because of lower regulatory ability than transcription factor *Lhy *or *Ccal*, *Elf3 *might play the same role as *Lhy *and *Ccal*. Just as expected, *Elf3 *contains glutamine-rich motif suggesting that it is a transcription factor [56].

Before entrance of the biological oscillator of the circadian system formed by *Toc1*, *Lhy*, and *Ccal*, a crucial gene of *Pif3 *[X_9_] (Figure 5) is mediated significantly by *PhyA *[X_7_] (-0.7631) and *PhyB *[X_11_] (0.1223) (see Eq.(9) in Table 1). This is consistent with the post-transcriptional interactions of Pif3-PhyA and Pif3-PhyB complexes. As a core gene in the biological oscillator, *Toc1 *[X_13_] is transcriptionally regulated by *Lhy *[X_3_] (0.7009) and *Ccal *[X_4_] (-1.4704) whilst *Pif3 *[X_16_] (-0.1698) is presumably considered as the bridge between *Toc1 *and phytochrome (Eq.(13), Table 1). From *Lhy *and *Ccal *point of view, they are both positively affected simultaneously by *Pif3 *implying the regulation on the transcriptional level [57]. In addition, *Toc1 *inhibits both *Lhy *and *Ccal *to form the structure of mutual transcriptional regulation (please compare Eqs.(3), (4) with Eq.(13) in Table 1). So we conclude that *Lhy *and *Ccal *function as principal transcription factors.

We also infer some downstream pathways of *Chs *[X_5_] (CHALCONE SYNTHASE), *Pap1 *[X_1_], and *Co *[X_2_] (CONSTANS) in Figure 5. *Chs *is known as correlated with UV-B protection. It seems that *Ccal *and *Lhy *have greater effect (2.7078, -0.7631, respectively) on *Chs *than *Pap1 *(-0.0455) as a transcription factor (Eq.(5) in Table 1). This might mean that *Chs *is regulated by *Pap1 *in a small scale with amplifying effect on the *cis*-regulatory level. *Co *is recognized as a pivotal gene of photoperiodic regulation of flowering. Indeed, strong regulations from *Ccal *and *Lhy *are identified to show that *Co *is regulated with a large-scale attenuation effect on the *cis*-regulatory level (Eq.(2) in Table 1).

**Figure 7:**
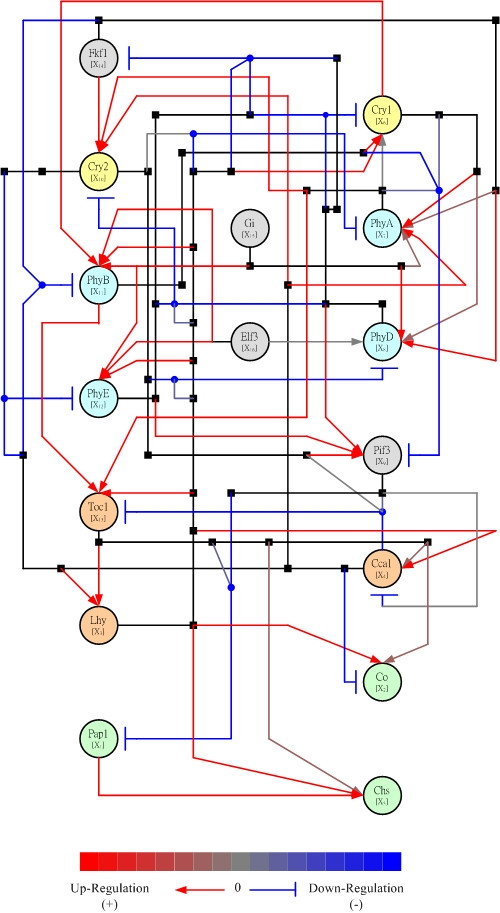
**The pathway of circadian regulatory system of Arabidopsis thaliana in Fig. 5 is repeated as independent validation by randomly reshuffling the time order of microarray experiment but with the same choices of target and regulatory genes. Obviously, the proposed pathway in Fig. 5 is destroyed by the reshuffling of experimental data**. The related genes are represented as ovals with different colors of light yellow (crytochrome), light blue (phytochrome), orange (biological clock genes), light green (some physiologically light-dependent downstream genes), and gray (other relevant genes not as target genes). There are three types of lines with colors of red (activation), blue (repression), and black. In addition to the black line representing the signal pipes from genes, the red lines are shown depending on the degree of activation whilst the blue lines are shown depending on the degree of repression. The black square symbol attached on the lines is the bifurcate node from the same pipe of signal. The circle symbol attached on the lines is the collecting nodes from different signal sources. The colored degree bar between activation and repression is also shown in the bottom of the figure.

In the overview of the circadian system in Figure 5, most red lines of activating regulation are found in the photo-transduction pathway between phytochrome (light blue ovals) and crytochrome (light yellow ovals) implying the chain interactions after the external light input. By the feedback regulations of *Lhy *and *Ccal *(orange ovals), represented by black lines with more linking to upstream genes, the photo-transduction pathways are stabilized to provide oscillation. On the other hand, more blue lines of inhibitive interactions are revealed in the biological-clock regulatory pathways relevant to *Co*, *Pap1*, and *Chs *(light green ovals) underlying the anti-phase functional regulation between these output pathways and the oscillator. In addition, the essential signal transduction factors of *Fkf1*, *Gi*, *Elf3*, and *Pif3 *(gray ovals) make some critical links between the functional blocks mentioned above in the circadian system[58]. Finally, in order to validate the proposed approach, an independent validation is also given by randomly reshuffling the time order of microarray experiment [see Additional file 2] but with the same choices of target gene and regulatory genes, as shown in Figure 7. It is seen that the proposed circadian regulatory pathway in Figure 5 is destroyed by reshuffling the experimental data.

#### b. Metabolic shift pathway

Sugars, such as glucose and sucrose, are excellent carbon sources for yeasts and almost all of the energy requirements of the cell can be satisfied by glycolysis [6,45,59-61,63-66]. *Saccharomyces cerevisiae *can switch from fermentatioon at high levels of glucose to respiration at low levels of glucose with major changes in metabolic activity (diauxic shift). In their experiment on the diauxic shift [30], DeRisi et al. inoculated cells from an exponentially growing culture into fresh medium and grew them at 30 for 21 hrs. This offers a resource to infer the possible allosteric regulation of enzymatic activities, protein modification and transcriptional regulation as shown in Table 2. In addition, the scheme of the corresponding inferred pathway is shown in Figure 6. In the overview of the inferring relationships in Table 2, the gluconeogenesis from *Pyk1 *to *pgm2 *and the partial fermentation from *Pyk1 *(PYRUVATE KINASE 1) to *Adh1 *and *Adh2 *(ETHANOL DEHYDROGENASE ISOZYME 1, 2) are unraveled as a result of the diauxic shift, so two sub-pathways in opposite directions are concluded.

In the fermentation direction, *Pykl *[Y_4_] encoding an enzyme, which catalyzes PEP (Phosphoenolpyruvate) to pyruvate, is negatively regulated (-5.8763) by *Pck1 *[Y_26_] (Eq.(4) in Table 2). *Pck1 *could be intepreted here as an indirect upstream transcription factor or regulatory gene for *Pyk1 *due to its function of decarboxylation and phosphorylation of oxalacetat in the presence of a nucleoside triphosphate and a divalent metal ion to yield PEP. Another *Gcrl *[Y_15_] gene is also identified as the strongest positive regulation (5.9829) to *Pyk1 *(also see Eq.(4) in Table 2), which is putatively considered as a transcription factor. This candidate transcription factor *Gcrl *of *Pdc1 *[Y_6_] (PYRUVATE DECARBOXYLASE ISOZYME 1) plays a more essential role (-2.5615, Eq.(6) in Table 2) than *Rap1 *[Y_17_] (0.1164), and *Pyk1 *[Y_4_] is an upstream regulatory factor coding an enzyme with the most positive effect (3.1295) on *Pdc1 *according to the production of acetaldehyde from pyruvate. In the last kernel of the fermentation, *Adh1 *[Y_7_] and *Adh2 *[Y_3_] are involved in the ethanol metabolism of carbohydrate storage. *Adh2 *is implicated to up-regulate *Adh1 *(0.5145, Eq.(7) in Table 2) under the catabolism from ethanol to acetaldehyde and is significantly up-regulated by *Adh1 *(1.0746, Eq.(3) in Table 2) to produce ethanol reversely. The mutual regulations of these two isozymes are within a tiny activation delay of 0.5-hr implying their close relationship. In addition, *Gcr2 *[Y_16_] and *Sfp1 *[Y_30_] with consistently dominant negative influences on *Adh2 *and *Adh1 *respectively would be at the transcriptional level presumably (see Eqs.(3), (7) in Table 2).

In the sub-pathway of glyconeogenesis, *Eno2 *[Y_2_] (ENOLASE ISOZYME 2) is regulated by *Pck1 *[Y_26_] (-0.7195) in the same way as *Pyk1 *while the main transcription factor is *Stp2 *[Y_31_] with significantly positive regulation (0.2147, see Eq.(2) in Table 2). As seen in Table 2, a causal cascade of *Eno2, Gpm1 *[Y_8_] (PHOSPHOGLYCERATE MUTASE l), Tpi1 [Y_10_] (TRIOSE-P ISOMERASE 1), Fba1 [Y_11_] (ALDOLASE 1), and Pgi1 [Y_9_] (PHOSPHOGLUCOSE ISOMERASE 1) indicates the construction of a trunk of the glyconeogenesis (see Eqs.(8), (9), (10), and (11) in Table 2). Among them, *Rap1 *[Y_17_] and *Gcrl *[Y_15_] are the common regulators of *Gpm1*, *Pgi1*, and *Tpi1*. This means that *Rap1 *and *Gcrl *might be the most important regulators in the glyconeogenesis pathway by the transcriptional binding. Finally, *Pgm2 *[Y_5_] (PHOSPHOGLUCOMUTASE 2) co-regulated by *Glk1 *[Y_27_] (GLUCOKINASE 1), *Hxk1 *[Y_28_], and *Hxk2 *[Y_29_] (HEXOKINASE 1, 2) significantly confers another pathway leading to the synthesis of UDP-GLU from Glucose-6-P (see Eq.(5) in Table 2).

**Figure 8:**
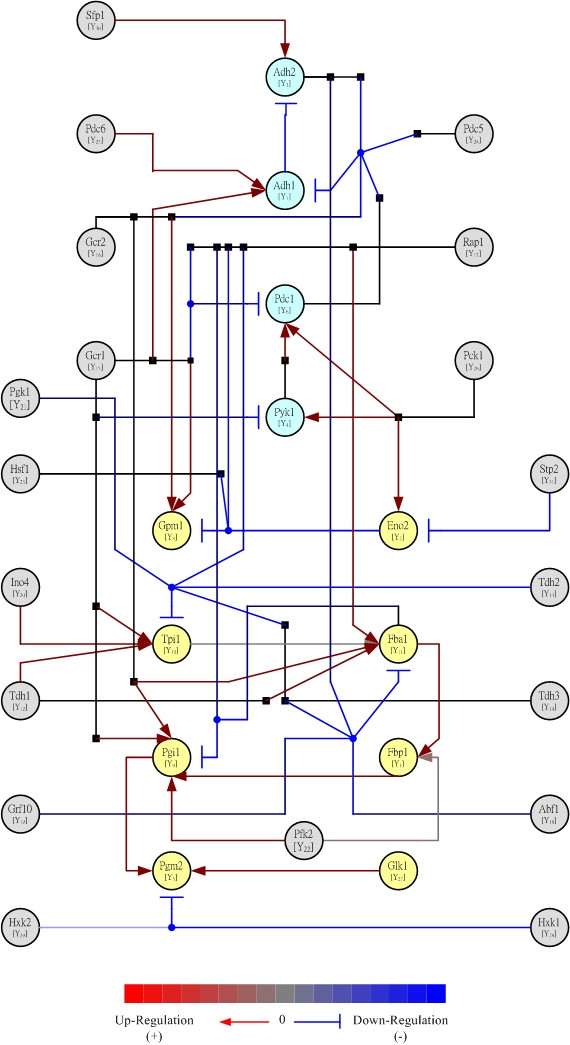
**The pathway of metabolic shift regulatory system of yeast Saccharomyces cerevisiae in Fig. 6 is repeated as independent validation by randomly reshuffling the time order of microarray experiment but with the same choices of target and regulatory genes. Obviously, the proposed pathway in Fig. 6 is destroyed by the reshuffling of experimental data**. The related genes are represented as ovals with different colors of light blue (fermentation), light yellow (gluconeogenesis), and gray (other relevant genes not as target genes). There are three types of lines with colors of red (activation), blue (repression), and black. In addition to the black line representing the signal pipes from genes, the red lines are shown depending on the degree of activation whilst the blue lines are shown depending on the degree of repression. The black square symbol attached on the lines is the bifurcate node from the same signal source. And the circle symbol attached on the lines is the collecting nodes from different signal sources. The colored degree bar between activation and repression is also shown in the bottom of the figure.

In the overview of the metabolic shift pathway in Figure 6, extremely significant regulations (vivid red lines or blue lines) from most transcription factors (gray ovals) means that transcriptional regulations are feasibly identified. However, in the fermentation sub-pathway (light blue ovals), the mutual regulations between *Adh1 *and *Adh2 *are apparent when compared with the obscure relationships in glyconeogenesis (light yellow ovals). Interestingly, three transcription factors *Gcr1, Gcr2 *and *Rap1 *(black-line signals) appear to have very significant effects on the metabolic shift pathway. Finally, in order to validate the proposed method, an independent validation is also given by randomly reshuffling the time order of microarray experiment but with same choices of target gene and regulatory gene, as shown in Figure 8. Obviously, the proposed metabolic shift in Figure 6 is destroyed by reshuffling the experimental data.

## Conclusion

Microarray expression analysis by the dynamic system approach offers an opportunity to generate functional regulation interpretation on the genome-wide scale. The crucial ontology behind using dynamic system techniques is that the causality between gene expression profiles could be identified according to the differential equation underlying a dynamic system. Therefore, because the microarray data were harvested with time progression, the simultaneously varied gene expressions implicated in a genetic regulatory system would be detected to infer the regulatory pathways in spite of the versatile interactions such as transcriptional control, protein phosphorylation, or specific enzyme regulation.

The clustering method answers the problem of what is the functional catalogue of a specific gene by the identification of resembling patterns of gene expressions. Similarly, the co-regulations of upstream genes in our method also imply their concurrent functions. In contrast to the clustering algorithm, the causality of time-course data has been smoothly drawn by our dynamic method. The Bayesian networks were used merely for forward probabilistic estimation with time transition lacking in the feedback linkages. This unidirectional problem would not happen in our algorithm. Owing to the quantitative regulatory abilities of our model, we have a greater diversity of regulatory influence than the Boolean networks, which are deterministic with merely two states.

In our dynamic system approach, we not only can link qualitatively the upstream genes to the downstream ones iteratively, but also indicate quantitatively their regulatory relationships, including the regulatory abilities and the activation delays. In terms of the regulatory abilities, the comparison between the upstream regulatory genes of a target gene can inspire us to ask which one is significant biologically and whether it is a positive or negative influence on the investigated gene. Moreover, the speculation of activation delays benefits the empirical reference by providing us when the upstream regulatory genes might interact with their target genes. Since any gene can be considered as a target gene to trace back its upstream regulators, these regulators are then considered as target genes to trace back their upstream regulators. Iteratively, the genetic regulatory pathway (or network) can be constructed to the genome-wide. According to the qualitative and quantitative features imbedded, two regulatory pathway examples are characterized as in Figure 5 and Figure 6 for the identification of the proposed method. In addition, using the Akaike Information Criterion (AIC), a proper number of regulatory genes would be affirmed. As a result, many links overlap with well-known regulatory and signaling pathways in the previous literature and several putative ones are also found. Furthermore, the activation or repression relationships inferred via the microarray data would distinctly uncover the overall effect of regulatory interactions among casual genes in pathways on the transcriptional level.

In the two pathways under investigation, we have a more detailed understanding about the regulatory interactions among phytochrome, crytochrome and biological clock in the circadian regulatory system. On the other hand, the sophisticated knowledge of the metabolic pathway after the diauxic shift can be unfolded properly in our analysis. Furthermore, the independent validation of our approach is also given by randomly reshuffling the time order of microarray experiment. We found that the proposed pathways in Figures 5 and 6 are all destroyed as shown in Figures 7 and 8, respectively. The successful analysis of these two pathways implies the development of a valid and high-throughput method. All of the programs have been released [see Additional file 1]

There are some shortcomings in our study. First, although the time-course microarray data are available, its lower samplings will distort the real changes of gene expressions, especially for quick dynamic evolution. A more sampling experiment with respect to the intrinsic turnover rate is expected to have more precise analysis. Secondly, a regulatory gene with larger activation delay would not be recognized because the less activation delay criterion is used, but this might be overcome by properly relaxing the criterion. Thirdly, activation profiles under the proteome should be highly correlated with the transcriptional profiles to elevate the interpretation of our system model. In general, the synchronous time-course microarray assay is more suitable to underlie the transcriptional binding among causal genes, but an inference of physical interactions in the post-transcriptional level also has sufficient feasibility in our study.

In the near future, the most pressing task is to investigate our presumed paths in the laboratory. As the pathway construction algorithms are further developed, we expect this system approach to have immense impact in elucidating the underlying molecular mechanisms of pathways in a variety of organisms, especially after the maturation of the protein chips. Ultimately, we envision that biologists will perform routine pathway inference to seek some novel regulations and to identify the evolutionarily conserved links.

## Methods

### I. Detection of regulatory function Gi(t) in equation (1.2)

After the decomposition of *G*_*i*_(*t*) in equation (1.2), we substitute equation (1.2) into equation (1.1) to obtain the following dynamic equation for the expression profile of the *i*-th gene,



In the above dynamic equation, parameters *a*_*i*_, *b*_*i*_, *α*_*n*_, and *β*_*n *_should be estimated by the time profile of microarray data of the *i*-th gene, i.e. these parameters should be specified so that the simulating output *X*_*i*_(*t*) of the dynamic model in equation (3.1) should meet the empirical expression profile of the *i*-th gene. The least-squares estimation method is employed to solve this parameter estimation problem.

To make the dynamic model effective, the dynamic equation in (3.1) should meet the expression profile at all time points *t *= *t*_1_,…, *t*_*m *_and is then arranged in a vector differential form. Consequently, the vector differential form underlined in this equation is applied to *m *time points in order.



where , , and *m *denotes the number of time points.

In the next step, formula (3.2) can be translated into a differential matrix equation as follows,

*Y*_*i *_= *A*_*i*_Φ_*i *_+ *E*_*i *_    (3.3)

where , Φ_*i *_= [*a*_*i *_*b*_*i *_*α*_*0 *_*β*_*0 *_… *α*_*N *_*β*_*N*_]^*T*^, and  are in vector forms, while  is a matrix.

To estimate the relevant unknown parameters in Φ_*i*_, the least-squares method below is used to derive the optimal parameters estimation of ,



Actually, the modeling error could be concluded into *E*_*i *_as the noise of the gene-expression profile or of the microarray chips. So the consideration of modeling error makes equation (3.3) approach more the reality. By the way, in order to get accurate data of  and  from the expression profile of the target gene, the cubic spline should be employed to interpolate the time profile of the target gene. Furthermore, the choice of N is based on the tradeoff between the accuracy of approximation in (1.2) and the complexity of parameter estimation in (3.4). In this study N = 6 is chosen because these harmonics are enough to approximate regulation functions.

### II. Maximum likelihood Estimate of kinetic parameters Ω_*i *_in equation (2.4)

Maximum likelihood method for Ω_*i *_in equation (2.4) is given as follows:



The log-likelihood function for given *m *data points is then described by



The necessary condition for the maximum likelihood estimation of variance *σ*^2 ^is , by which equation (2.5) is obtained.

Substituting equation (2.5) into equation (4.2) yields,



meaning that we can find the maximum likelihood estimation of Ω_*i *_by minimizing the value of *σ*^2 ^in equation(2.5). Then, the maximum likelihood estimate in equation (2.6) is obtained by  in equation(2.5).

## Authors' contributions

W.C. Chang and Chang-Wei Li carried out the computational studies and analysis. B.S. Chen gave the topic and suggestions. All authors read and approved the final manuscript.

## Supplementary Material

Additional File 1 The raw data has been transform to .mat file, which is one of the Matlab files. This file has been divided into three parts: Name, Time, and Profile. [see Additional file 1] Name denotes the names of microarray data. Time denotes the time points of microarray data. Profile denotes the gene profiles of microarray data. Click here for file

Additional File 2 The 'Shuffled_data' is the Shuffled raw data. [see Additional file 2] Click here for file

## Figures and Tables

**Table 1:** The dynamic equation set of the identified upstream regulators and their regulatory relationships to the specific target genes in the pathway of circadian regulatory system of Arabidopsis thaliana.














Target Genes	X_1_	X_2_	X_3_	X_4_	X_5_	X_6_	X_7_	X_8_
	Papl*	Co	Lhy*	Ccal*	Chs	Cryl	PhyA	PhyD
	X_9_	X_10_	X_11_	X_12_	X_13_			
	Pif3	Cry2	PhyB	PhyE	Tocl*			
Other Genes	X_14_	X_15_	X_16_					
	Fkfl	Gi	Elf3					

**Table 2:** The dynamic equation set of the identified upstream regulators and their regulatory relationships to the specific target genes in the metabolic shift pathway of yeast Saccharomyces cerevisiae.












Target Genes	y_1_	Y_2_	Y_3_	Y_4_	Y_5_	Y_6_	Y_7_	Y_8_
	Fbpl	Eno2	Adh2	Pykl	Pgm2	Pdcl	Adhl	Gpm1
	Y_9_	Y_10_	Y_11_					
	Pgil	Tpil	Fbal					
Other Genes	Y_12_	Y_13_	Y_14_	Y_15_	Y_16_	Y_17_	Y_18_	Y_19_
	Tdhl	Tdh2	Tdh3	Gcrl*	Gcr2*	Rap1*	Abfl*	GrflO*
	Y_20_	Y_21_	Y_22_	Y_23_	Y_24_	Y_25_	Y_26_	Y_27_
	Ino4*	Pgkl	Pfk2	Hsfl*	Pdc5	Pdc6	Pckl	Glkl
	Y_28_	Y_29_	Y_30_	Y_31_				
	Hxkl	Hxk2	Sfpl*	Stp2*				
